# Supplementary material for: SARS-CoV-2 genomes from Saudi Arabia implicate nucleocapsid mutations in host response and increased viral load
Source: Nat Commun. 2022 Feb 1;13:601. doi: 10.1038/s41467-022-28287-8 (PMC8807822; doi:10.1038/s41467-022-28287-8)
Supplement: Supplementary file 1 — Supplementary Information [file 41467_2022_28287_MOESM1_ESM.pdf]

SARS-CoV-2 genomes from Saudi Arabia implicate nucleocapsid mutations in host response and increased viral load

Supplementary Information

Supplementary Note 1. Daily Cases in Saudi Arabia

The numbers of Covid-19 cases registered in Saudi Arabian Cities were collected from The Ministry of Health Command Centre for COVID-19 (https://covid19.moh.gov.sa), Saudi Center of Disease Control and Prevention (https://covid19.cdc.gov.sa/), the Saudi Press Agency (SPA) (https://www.spa.gov.sa/search.php?lang=en&search=COVID), The Saudi Ministry of Interior (https://www.moi.gov.sa/), and Algaissi *et al.*[1].

Supplementary Table S1. Cities providing samples

City	Hospital	Total samples	Samples with patient outcome information	Deceased patients	Samples with R203K/G204R SNPs	R203K/G204R frequency
Madinah	General	42	37	7	2	0.0476
	King Fahad Medical	16	14	6	0	0
	Madinah Children Hospital	3	3	0	0	0
	Ohoud	251	238	63	8	0.0319
Makkah	Al Zahir	16	16	3	3	0.1875
	Al Noor	71	69	0	0	0
	Quarantine Hotel	144	144	0	4	0.0278
Jeddah	King Abdullah Medical Complex	262	262	105	101	0.3855
Riyadh	Sulaiman Al Habib	69	53	15	6	0.0870
Eastern Region	Sulaiman Al Habib, Eastern	18	14	0	0	0
**TOTAL**		**892**	**850**	**199**	**124**	**0.1390**

Supplementary Table S2. Patient data and comorbidities

	Female	Male	No data available
Number of patients	195	682	15
Age range (years)	0-95	0-94	.
Median age (years)	50	50	.
Average age (years)	49.8	49.2	.

	Patients	Incidence	Mortality	Incidences reported in other studies[2-8]
Diabetes	272	0.39	0.34	0.074-0.431
Hypertension	238	0.35	0.35	0.095-0.375
Cardiovascular	76	0.11	0.45	
Lung BA	44	0.06	0.23	
Kidney CDK	24	0.03	0.49	0.05-0.189
Cerebrovascular	10	0.01	0.40	
Any comorbidity	386	0.56	0.32	
No comorbidity	303	0.44	0.24	

Notes: The patient samples without available sex information are also without age information. 203 patients have no available data on comorbidity, and these are not included in the incidence calculations. Only samples with available patient outcome information (850 samples) are included in the mortality calculations.

<u>**Supplementary Table S3. Detected indels**</u>

position	type	length	ref	allele	sample count
103	del	2	CTG	C	2
509	del	9	GGTCATGTTA	G	1
668	del	3	AGTT	A	3
685	del	9	AAAGTCATTT	A	4
898	ins	262	TTCATGCACTTTGTCCGAACAACTGGACTTTATTGACACTAAGAGGGGTGTATACTGCTGCCGTGAAC	TTCATGCACTTTGTCCGAACAACTGGACTTTATTGACACTAAGAGGGGTGTATACTGCTGCCGTGAACCACTTTTTCTTTGCATTTACTTTTTTATAGGAACTCCTGTCATCACTCTCTCACACACACACTTAGATGAACCTGATGGCTACCCTCTTGAGTGCATTAAAGACCTTCTAGCACGTGCTGGTAAAGCCTCATGCACTTTGTCCGAACAACTGGACTTTATTGACACTAAGAGGGGTGTATACTGCTGCCGTGAAC	1
2463	del	4	TAAAA	TAA	1
2628	del	3	TTAT	TT	1
2882	ins	282	TGTGTTGTGGCAGATGCTGTC	TGTGTTGTGGCAGATGCTGTCGTGTTGTGGCAGATGCTGTCGTGTTGTGGCAGATGCTGTCGTGTTGTGGCAGATGCTGTCGTGTTGTGGCAGAGGCTGCTCGTGTTGTGGCAGATGCTGTCGTGTTGTGGCAGATGCTGTCGTGTTGTGGCAGATGCTGTAGTGTTGCATCAGAGGCTGCTCGTGTTGTGGCAGATGCTGTCGTGTTGTGGCAGATGCTGTCGTGTTGTGGCAGATGCTGTCGTGTTGTGGCAGATGCTGTCGTGTTGTGGCAGATGCTGTC	1
2882	ins	141	TGTGTTGTGGCAGATGCTGTC	TGTGTTGTGGCAGATGCTGTCGTGTTGTGGC	1

				AGATGCTGTCGTGTTGTGGCAGATGCTGTCGTGTTGTGGCAGATGCTGTCGTGTTGTGGCAGATGCTGTCGTGTTGCATCAGAGGCTGCTCGTGTTGTGGCAGATGCTGTC	
7626	ins	49	ATTGTGATACATTCTGTGCTGGTAGT	ATTGTGATACATTCTGTGCTGGTAGTTGTGATACATTCTGTGCTGGTAGT	2
10535	del	27	TACATGCACCATATGGAATTACCAACTG	T	1
11074	ins	11	CTTTTTTTT	CTTTTTTTTTTT	1
18896	ins	309	TTGTTAAGCGTGTTGACTGGACTATTGAATATCCTATAATTGGTGATGAACTGAAGATTAATGCGGCTTGTAGAAAGGTTCAACA	TTGTTAAGCGTGTTGACTGGACTATTTAATATCCTATAATTGGTGATGAACTGAAGATTAATGCGGCTTGTAGAAAGGTTCAACATAACATGTTGTGCCAACCACCAGCACTCCTGGGACCTCCACAGTGCACCTGGCAACCTCTGGGACTCCATCCTCCCTGCCTGGCCACACAGCCCCTGTCCCTCTCTTGATACCATTCACCCTCAACTTTACCAGATGGGAATGTTAAGCGTGTTGACTGGACTATTGAATATCCTATAATTGGTGATGAACTGAAGATTAATGCGGCTTGTAGAAAGGTTCAACA	1
19517	del	3	TGTA	T	1
21066	del	5	TAAAAA	TAA	1
21561	del	2	CAA	CA	2
21624	del	33	GAACTCAATTACCCCCTGCATACACTAATTCTTT	G	1
21740	del	45	TCCAATGTTACTTGGTTCCATGCTATACATGTCTCTGGGACCAATG	TCCAATG	1
21781	ins	3	CAA	CAAA	1
21990	del	6	TTTATTA	TTTA	3
22048	del	3	TGCG	T	1
22288	del	6	TGCTTTA	T	1
22353	del	7	CTTATTAT	CTTAT	1

23701	del	1	CA	C	1
27263	del	29	CTTTTAAAGTTTCCATTTGGAATCTTGATT	CTT	1
27694	del	8	TTTCTTATT	TTT	1
27697	del	5	CTTATT	CTT	4
28949	del	11	AGATTGAACCAG	A	1
29727	del	22	TTTCACCGAGGCCACGCGGAGTA	T	1
29755	del	1	GA	G	2
29774	del	18	CTAGGGAGAGCTGCCTATA	CTA	3
29865	ins	5	AA	AAAACA	1
29865	ins	7	AA	AAACAACA	4
29865	ins	3	AA	AACA	1
29865	ins	9	AA	AACCACAACA	1
29865	ins	10	AA	AAGCCACAACA	1
29866	ins	3	A	AACC	1
29866	ins	6	A	AAGATGC	1
29866	ins	9	A	AAGCAGCCTC	1
29866	ins	3	A	AATG	1
29866	ins	8	A	AGCAGATGC	1

Supplementary Table S4. Relationship between Nextstrain clades and Pangolin lineages

Nextstrain clade	Pangolin lineages
19A	B; B.40
19B	A; A.1; A.2; A.3
20A	B.1; B.1.5
20A.EU1	B.1.177
20A.EU2	B.1.160; B.1.160.6
20B	B.1.1; D.2
20B/501Y.V1	B.1.1.7
20C	B.1; B.1.2
20C/501Y.V2	B.1.351

Note: Clade and lineage assignments were retrieved from all samples available at GISAID on December 31st, 2020. Pangolin lineages constituting at least 10% of the samples assigned to a given Nextstrain clade are included in this table.

Supplementary Table S5. Regression table for the association between R203K/G204R mutation and severity (ICU or deceased) adjusting for other mutations, sex, comorbidities, hospital and age.

Parameter	Log-Odds	SE	95% CI	z	p
(Intercept)	-2.55	0.98	[-4.46,-0.63]	-2.61	0.009
R203K/G204R	1.18	0.49	[0.22,2.13]	2.41	0.016
C241T	-1.85	0.92	[-3.65,-0.05]	-2.01	0.044
C1191T	-0.99	0.72	[-2.40,0.43]	-1.37	0.172
C3037T	0.26	0.37	[-0.48,0.99]	0.69	0.493
G10427A	34.49	6.71e+07	[-1.32e+08,1.32e+08]	5.14e-07	>.999
C14408T	2.40	1.03	[0.38,4.41]	2.33	0.020
C15352T	32.04	3.87e+07	[-7.59e+07,7.59e+07]	8.27e-07	>.999
C18877T	0.38	0.26	[-0.14,0.90]	1.45	0.147
A23403G	-0.32	0.27	[-0.84,0.20]	-1.19	0.234
G25563T	-0.38	0.49	[-1.33,0.58]	-0.77	0.440
C26735T	0.48	0.57	[-0.64,1.59]	0.84	0.403
T27484C	1.77	1.34	[-0.85,4.39]	1.32	0.186
C28139T	-1.61	7.50e+07	[-1.47e+08,1.47e+08]	-2.15e-08	>.999
sex[M]	0.13	0.25	[-0.36,0.61]	0.52	0.605
comorbidTRUE	0.39	0.23	[-0.05,0.84]	1.73	0.084
hospital[GEN-MED]	0.74	0.78	[-0.79,2.28]	0.95	0.344
hospital[KAMC]	1.05	0.68	[-0.28,2.39]	1.55	0.122
hospital[KFMH]	1.44	0.96	[-0.43,3.32]	1.51	0.131
hospital[MCH]	-30.06	4.75e+07	[-9.30e+07,9.30e+07]	-6.34e-07	>.999
hospital[NOOR]	-2.04	1.00	[-4.00,-0.08]	-2.04	0.041
hospital[OHOUD]	1.39	0.68	[0.05,2.73]	2.03	0.043
hospital[SULAIMANAHABIB]	4.51	1.23	[2.10,6.92]	3.67	<.001
hospital[SULAIMANALHABIB_eastern]	0.44	0.92	[-1.37,2.25]	0.48	0.632

Parameter	z	df	p
Smooth term (age)	53.60	1.00	<.001

Note: For each of the parameters, the table shows the regression coefficient estimate, standard error, and 95% confidence interval, and the two-sided z test and p-value testing the null hypothesis that the coefficient is equal to 0.

Supplementary Table S6. Regression table for the association between R203K/G204R mutations and severity (ICU or deceased) adjusting for other mutations, sex, comorbidities, hospital, age and time.

Parameter	Log-Odds	SE	95% CI	z	p
(Intercept)	-1.50	1.02	[-3.50,0.50]	-1.47	0.142
R203K/G204R	1.38	0.56	[0.28,2.48]	2.46	0.014
C241T	-2.46	1.02	[-4.46,-0.46]	-2.41	0.016
C1191T	-1.29	0.83	[-2.91,0.33]	-1.57	0.117
C3037T	0.35	0.42	[-0.47,1.18]	0.85	0.396
G10427A	34.66	6.71e+07	[-1.32e+08,1.32e+08]	5.16e-07	>.999
C14408T	1.46	1.07	[-0.63,3.55]	1.37	0.171
C15352T	32.65	3.87e+07	[-7.59e+07,7.59e+07]	8.43e-07	>.999
C18877T	0.72	0.30	[0.12,1.31]	2.37	0.018
A23403G	0.15	0.32	[-0.48,0.77]	0.46	0.644
G25563T	-0.38	0.58	[-1.50,0.75]	-0.65	0.514
C26735T	0.60	0.67	[-0.72,1.91]	0.89	0.375
T27484C	0.94	1.41	[-1.81,3.70]	0.67	0.503
C28139T	0.15	7.50e+07	[-1.47e+08,1.47e+08]	1.97e-09	>.999
sex[M]	0.28	0.28	[-0.27,0.83]	1.00	0.318
comorbidTRUE	0.27	0.24	[-0.21,0.74]	1.11	0.266
hospital[GEN-MED]	1.35	0.88	[-0.37,3.07]	1.54	0.123
hospital[KAMC]	0.82	0.71	[-0.57,2.22]	1.16	0.248
hospital[KFMH]	1.98	0.99	[0.03,3.93]	1.99	0.046
hospital[MCH]	-33.79	4.75e+07	[-9.30e+07,9.30e+07]	-7.12e-07	>.999
hospital[NOOR]	-1.98	1.08	[-4.10,0.13]	-1.84	0.066
hospital[OHOUD]	0.86	0.74	[-0.60,2.31]	1.15	0.249
hospital[SULAIMANALHABIB]	4.16	1.51	[1.19,7.13]	2.74	0.006
hospital[SULAIMANALHABIB_eastern]	0.24	0.92	[-1.57,2.05]	0.26	0.796

Parameter	z	df	p
Smooth term (age)	36.72	1.00	<.001
Smooth term (sampling_time)	52.41	7.01	<.001

Note: For each of the parameters, the table shows the regression coefficient estimate, standard error, and 95% confidence interval, and the two-sided z test and p-value testing the null hypothesis that the coefficient is equal to 0.

Supplementary Table S7. Regression table for the association between R203K/G204R mutations and mortality (deceased) adjusting for other mutations, sex, comorbidities, hospital and age.

Parameter	Log-Odd	SE	95% CI	z	p
(Intercept)	-2.07	0.95	[-3.94,-0.21]	-2.18	0.029
R203K/G204R	1.04	0.45	[0.16,1.92]	2.31	0.021
C241T	-1.44	0.84	[-3.09,0.21]	-1.71	0.088
C1191T	-0.74	0.71	[-2.13,0.65]	-1.04	0.297
C3037T	-0.12	0.34	[-0.79,0.56]	-0.34	0.735
G10427A	43.28	6.71e+0	[-1.32e+08,1.32e+08	6.45e-07	>.99
C14408T	1.26	0.97	[-0.64,3.17]	1.30	0.193
C15352T	0.47	0.99	[-1.48,2.41]	0.47	0.637
C18877T	0.34	0.24	[-0.14,0.81]	1.38	0.166
A23403G	-0.45	0.23	[-0.90,0.00]	-1.95	0.051
G25563T	-0.15	0.47	[-1.08,0.77]	-0.33	0.742
C26735T	0.50	0.54	[-0.55,1.56]	0.94	0.349
T27484C	1.18	0.98	[-0.74,3.10]	1.20	0.229
C28139T	-41.79	6.71e+0	[-1.32e+08,1.32e+08	-6.23e-0	>.99
sex[M]	0.58	0.25	[0.09,1.07]	2.33	0.020
comorbidTRUE	0.18	0.21	[-0.23,0.59]	0.86	0.389
hospital[GEN-MED]	0.18	0.83	[-1.45,1.81]	0.21	0.831
hospital[KAMC]	0.70	0.71	[-0.69,2.10]	0.99	0.322
hospital[KFMH]	1.23	0.96	[-0.64,3.11]	1.29	0.196
hospital[MCH]	-39.31	3.87e+0	[-7.59e+07,7.59e+07	-1.01e-0	>.99
hospital[NOOR]	-41.24	8.14e+0	[-1.60e+07,1.60e+07	-5.07e-0	>.99
hospital[OHOUD]	0.48	0.71	[-0.92,1.88]	0.68	0.499
hospital[QRTN-MK]	-40.90	5.61e+0	[-1.10e+07,1.10e+07	-7.29e-0	>.99
hospital[SULAIMANALHABIB]	0.15	0.77	[-1.36,1.67]	0.20	0.844
hospital[SULAIMANALHABIB_eastern	-41.16	1.86e+0	[-3.65e+07,3.65e+07	-2.21e-0	>.99

Parameter	z	df	p
Smooth term (age)	37.74	1.00	<.001

Note: For each of the parameters, the table shows the regression coefficient estimate, standard error, and 95% confidence interval, and the two-sided z test and p-value testing the null hypothesis that the coefficient is equal to 0.

Supplementary Table S8. Regression table for the association between R203K/G204R mutations and mortality (deceased) adjusting for other mutations, sex, comorbidities, hospital, age and time.

Parameter	Log-Odds	SE	95% CI	z	p
(Intercept)	-2.09	1.03	[-4.11,-0.08]	-2.03	0.042
R203K/G204R	0.58	0.50	[-0.41,1.56]	1.15	0.249
C241T	-1.55	0.86	[-3.23,0.13]	-1.80	0.071
C1191T	-0.90	0.78	[-2.43,0.63]	-1.16	0.247
C3037T	-0.17	0.38	[-0.91,0.58]	-0.44	0.661
G10427A	28.68	1.14e+06	[-2.23e+06,2.23e+06]	2.53e-05	>.999
C14408T	0.74	0.99	[-1.20,2.68]	0.75	0.454
C15352T	-0.48	1.04	[-2.53,1.56]	-0.46	0.645
C18877T	0.40	0.27	[-0.12,0.92]	1.50	0.133
A23403G	0.22	0.26	[-0.29,0.73]	0.84	0.399
G25563T	-0.14	0.52	[-1.15,0.87]	-0.27	0.783
C26735T	0.14	0.59	[-1.01,1.30]	0.24	0.807
T27484C	0.62	0.99	[-1.32,2.57]	0.63	0.529
C28139T	-27.90	1.14e+06	[-2.23e+06,2.23e+06]	-2.46e-05	>.999
sex[M]	0.73	0.27	[0.19,1.27]	2.67	0.008
comorbidTRUE	0.16	0.22	[-0.28,0.59]	0.70	0.482
hospital[GEN-MED]	0.84	0.92	[-0.97,2.65]	0.91	0.363
hospital[KAMC]	0.74	0.75	[-0.72,2.21]	0.99	0.321
hospital[KFMH]	1.88	1.01	[-0.10,3.85]	1.86	0.062
hospital[MCH]	-39.67	3.87e+07	[-7.59e+07,7.59e+07]	-1.02e-06	>.999
hospital[NOOR]	-40.77	8.14e+06	[-1.60e+07,1.60e+07]	-5.01e-06	>.999
hospital[OHOUD]	0.30	0.76	[-1.20,1.80]	0.39	0.696
hospital[QRTN-MK]	-40.17	5.61e+06	[-1.10e+07,1.10e+07]	-7.16e-06	>.999
hospital[SULAIMANALHABIBHABIB]	0.40	0.86	[-1.29,2.09]	0.47	0.641
hospital[SULAIMANALHABIB_eastern]	-41.16	1.86e+07	[-3.65e+07,3.65e+07]	-2.21e-06	>.999

Parameter	F	df	p
Smooth term (age)	31.51	1.00	<.001
Smooth term (sampling_time)	47.95	5.10	<.001

Note: For each of the parameters, the table shows the regression coefficient estimate, standard error, and 95% confidence interval, and the two-sided z test and p-value testing the null hypothesis that the coefficient is equal to 0.

Supplementary Table S9. Regression table for the association between R203K/G204R mutations and viral load (copynumber_N1) adjusting for qPCRkit, other mutations, sex, comorbidities, hospital, age and time.

Parameter	Coefficient	SE	95% CI	t	p
(Intercept)	5.62	0.65	[4.34,6.91]	8.59	<.001
R203K/G204R	1.33	0.31	[0.72,1.93]	4.30	<.001
qPCRkit[taqpath]	1.19	0.31	[0.59,1.80]	3.89	<.001
C241T	0.57	0.46	[-0.34,1.47]	1.23	0.221
C1191T	0.42	0.51	[-0.58,1.43]	0.82	0.410
C3037T	-0.61	0.25	[-1.09,-0.13]	-2.47	0.014
G10427A	-0.55	1.89	[-4.25,3.15]	-0.29	0.771
C14408T	-1.47	0.53	[-2.50,-0.43]	-2.77	0.006
C15352T	0.48	0.88	[-1.25,2.21]	0.55	0.585
C18877T	0.13	0.16	[-0.19,0.44]	0.79	0.429
A23403G	0.38	0.17	[0.05,0.72]	2.25	0.025
G25563T	-0.52	0.26	[-1.04,0.00]	-1.97	0.049
C26735T	1.39	0.28	[0.83,1.94]	4.92	<.001
T27484C	1.01	0.74	[-0.44,2.47]	1.37	0.172
C28139T	0.86	2.05	[-3.17,4.89]	0.42	0.676
sex[M]	-0.16	0.17	[-0.49,0.17]	-0.95	0.342
comorbidTRUE	0.02	0.15	[-0.29,0.32]	0.10	0.920
hospital[GEN-MED]	-1.61	0.66	[-2.90,-0.32]	-2.45	0.015
hospital[KAMC]	-1.21	0.55	[-2.29,-0.14]	-2.22	0.027
hospital[KFMH]	-0.83	0.72	[-2.25,0.59]	-1.15	0.251
hospital[MCH]	-2.52	1.28	[-5.03,-0.01]	-1.97	0.049
hospital[NOOR]	-1.92	0.61	[-3.11,-0.72]	-3.15	0.002
hospital[OHOUD]	-0.90	0.56	[-2.00,0.20]	-1.61	0.108
hospital[QRTN-MK]	-3.32	0.61	[-4.52,-2.13]	-5.47	<.001
hospital[SULAIMANALHABIB]	-1.42	0.64	[-2.68,-0.17]	-2.23	0.026
hospital[SULAIMANALHABIB_eastern]	-2.47	0.73	[-3.90,-1.04]	-3.39	<.001

Parameter	F	df	p
Smooth term (age)	2.63	2.46	0.046
Smooth term (sampling_time)	4.89	8.02	<.001

Note: For each of the parameters, the table shows the regression coefficient estimate, standard error, and 95% confidence interval, and the two-sided t test and p-value testing the null hypothesis that the coefficient is equal to 0.

Supplementary Table S10. Primers used in the study

Primers used for cloning

**Primer Name**	**Sequence 5' to 3'**
pLVX-N1-F1	CTATTTCCGGTGAATTCGCCG
pLVX-N1-R1	GGGGCGGGATCCTTACTTTTC
pLVX-N1-Mut-F1	CCAGGGTCCAGTAAACGAACAAGTCCGGCGC
pLVX-N1-Mut-R1	GCGCCGGACTTGTTCGTTTACTGGACCCTGG

**Primers and Probe for SARS-CoV-2**	
**Name**	**Catalog#**
nCOV_N1 Forward Primer Aliquot, 50 nmol	10006821
nCOV_N1 Reverse Primer Aliquot, 50 nmol	10006822
nCOV_N1 Probe Aliquot, 25 nmol	10006823
nCOV_N2 Forward Primer Aliquot, 50 nmol	10006824
nCOV_N2 Reverse Primer Aliquot, 50 nmol	10006825
nCOV_N2 Probe Aliquot, 25 nmol	10006826
E gene E_Sarbeco_F	ACAGGTACGTTAATAGTTAATAGCGT
E_Sarbeco_R	ATATTGCAGCAGTACGCACACA
E_Sarbeco_P1	FAM-ACACTAGCCATCCTTACTGCGCTTCG-BBQ
2019-nCoV (ORF1ab) (FAM dye)	TaqMan™ 2019-nCoV Assay Kit v1 (Cat. No. A47532)
2019-nCoV (S Gene) (FAM dye)	TaqMan™ 2019-nCoV Assay Kit v1 (Cat. No. A47532)

Supplementary Table S11. Datasets associated with the MaxQuant analysis

Experiment	Target Protein AP-MS	Replicates	Technical Replicates	Raw Data File Designation (submitted to ProteomXchange)
Mock AP-MS	Used as negative control AP-MS	1	NA	20210701_C1.raw
		2	NA	20210701_C2.raw
		3	NA	20210701_C3.raw
N protein wildtype AP-MS	Used for differential interaction analysis	1	NA	N_wildtype_01.raw
		2	NA	N_wildtype_02.raw
		3	NA	N_wildtype_03.raw
N protein KR-mutant AP-MS	Used for differential interaction analysis	1	NA	N_mutant_01.raw
		2	NA	N_mutant_02.raw
		3	NA	N_mutant_03.raw
N protein wildtype AP-MS	Used for phosphorylation detection analysis	1	1	20210108_WT1_a.raw
			2	20210108_WT1_b.raw
			3	20210108_WT1_c.raw
		2	1	20210108_WT2_a.raw
			2	20210108_WT2_b.raw
			3	20210108_WT2_c.raw
		3	1	20210108_WT3_a.raw
			2	20210108_WT3_b.raw
			3	20210108_WT3_c.raw
		4	1	20210108_WT4_a.raw
			2	20210108_WT4_b.raw

			3	20210108_WT4_c.raw
		5	1	20210108_WT5_a.raw
			2	20210108_WT5_b.raw
			3	20210108_WT5_c.raw
**N protein KR-mutant AP-MS**	**Used for phosphorylation detection analysis**	1	1	20210108_M1_a.raw
			2	20210108_M1_b.raw
			3	20210108_M1_c.raw
		2	1	20210108_M2_a.raw
			2	20210108_M2_b.raw
			3	20210108_M2_c.raw
		3	1	20210108_M3_a.raw
			2	20210108_M3_b.raw
			3	20210108_M3_c.raw
		4	1	20210108_M4_a.raw
			2	20210108_M4_b.raw
			3	20210108_M4_c.raw
		5	1	20210108_M5_a.raw
			2	20210108_M5_b.raw
			3	20210108_M5_c.raw

NA = Not Applicable

Supplementary References

1 Algaissi, A. A., Alharbi, N. K., Hassanain, M. & Hashem, A. M. Preparedness and response to COVID-19 in Saudi Arabia: Building on MERS experience. *J Infect Public Health* **13**, 834-838, doi:10.1016/j.jiph.2020.04.016 (2020).

2 Barbu, M. G., Thompson, R. J., Thompson, D. C., Cretoiu, D. & Suciu, N. The Impact of SARS-CoV-2 on the Most Common Comorbidities-A Retrospective Study on 814 COVID-19 Deaths in Romania. *Front Med (Lausanne)* **7**, 567199, doi:10.3389/fmed.2020.567199 (2020).

3 Reilev, M. *et al.* Characteristics and predictors of hospitalization and death in the first 11 122 cases with a positive RT-PCR test for SARS-CoV-2 in Denmark: a nationwide cohort. *Int J Epidemiol* **49**, 1468-1481, doi:10.1093/ije/dyaa140 (2020).

4 Yang, J. *et al.* Prevalence of comorbidities and its effects in patients infected with SARS-CoV-2: a systematic review and meta-analysis. *Int J Infect Dis* **94**, 91-95, doi:10.1016/j.ijid.2020.03.017 (2020).

5 Espinosa, O. A. *et al.* Prevalence of comorbidities in patients and mortality cases affected by SARS-CoV2: a systematic review and meta-analysis. *Rev Inst Med Trop Sao Paulo* **62**, e43, doi:10.1590/S1678-9946202062043 (2020).

6 Zhou, F. *et al.* Clinical course and risk factors for mortality of adult inpatients with COVID-19 in Wuhan, China: a retrospective cohort study. *Lancet* **395**, 1054-1062, doi:10.1016/S0140-6736(20)30566-3 (2020).

7 Pantea Stoian, A. *et al.* Death by SARS-CoV 2: a Romanian COVID-19 multi-centre comorbidity study. *Scientific reports* **10**, 21613, doi:10.1038/s41598-020-78575-w (2020).

8 Li, B. *et al.* Prevalence and impact of cardiovascular metabolic diseases on COVID-19 in China. *Clin Res Cardiol* **109**, 531-538, doi:10.1007/s00392-020-01626-9 (2020).

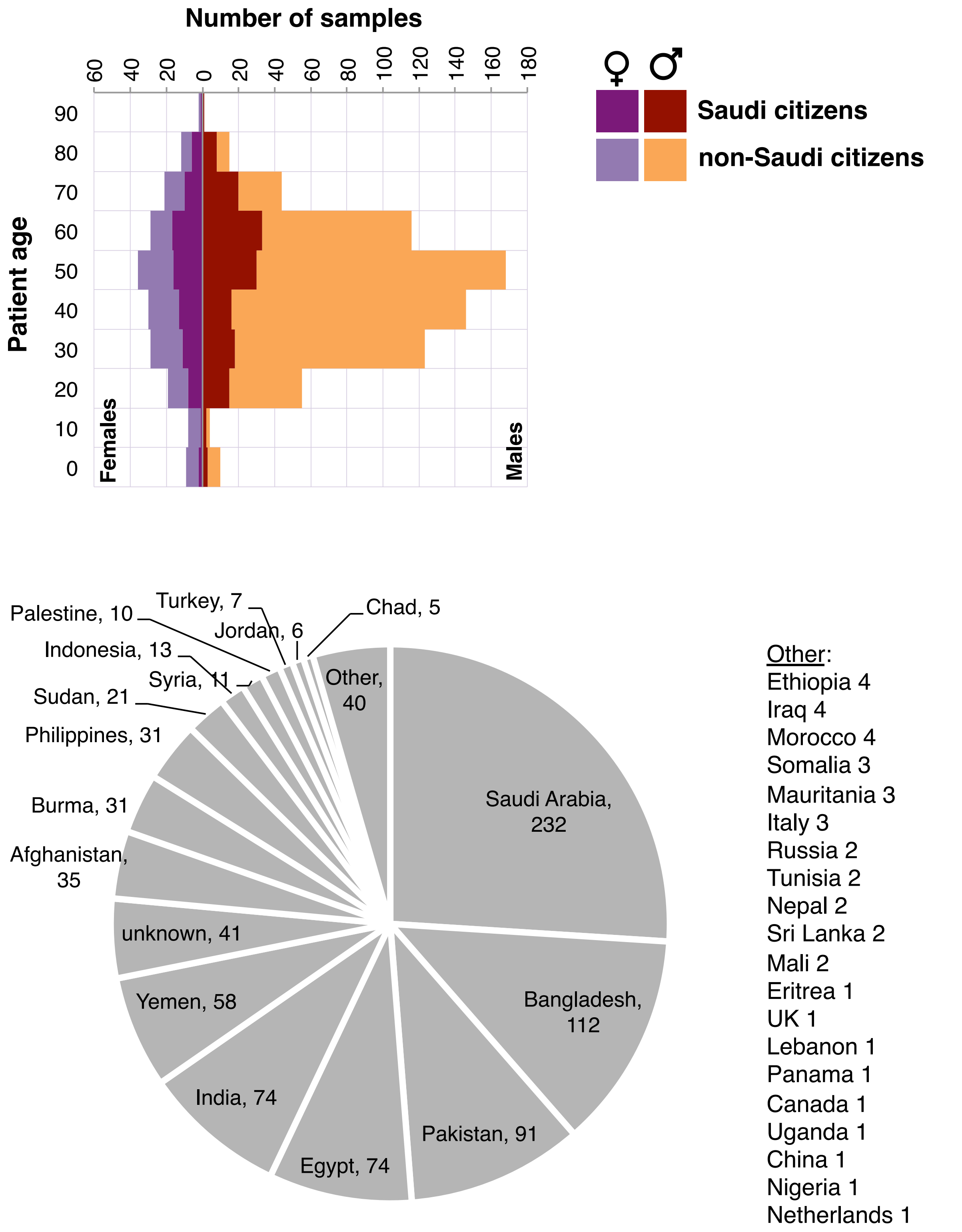

Supplementary Figure S1
Top: Patient age and gender shown for Saudi and non-Saudi citizens.
Bottom: Nationalities of patients. Numbers denote the total number of patients with a given nationality.

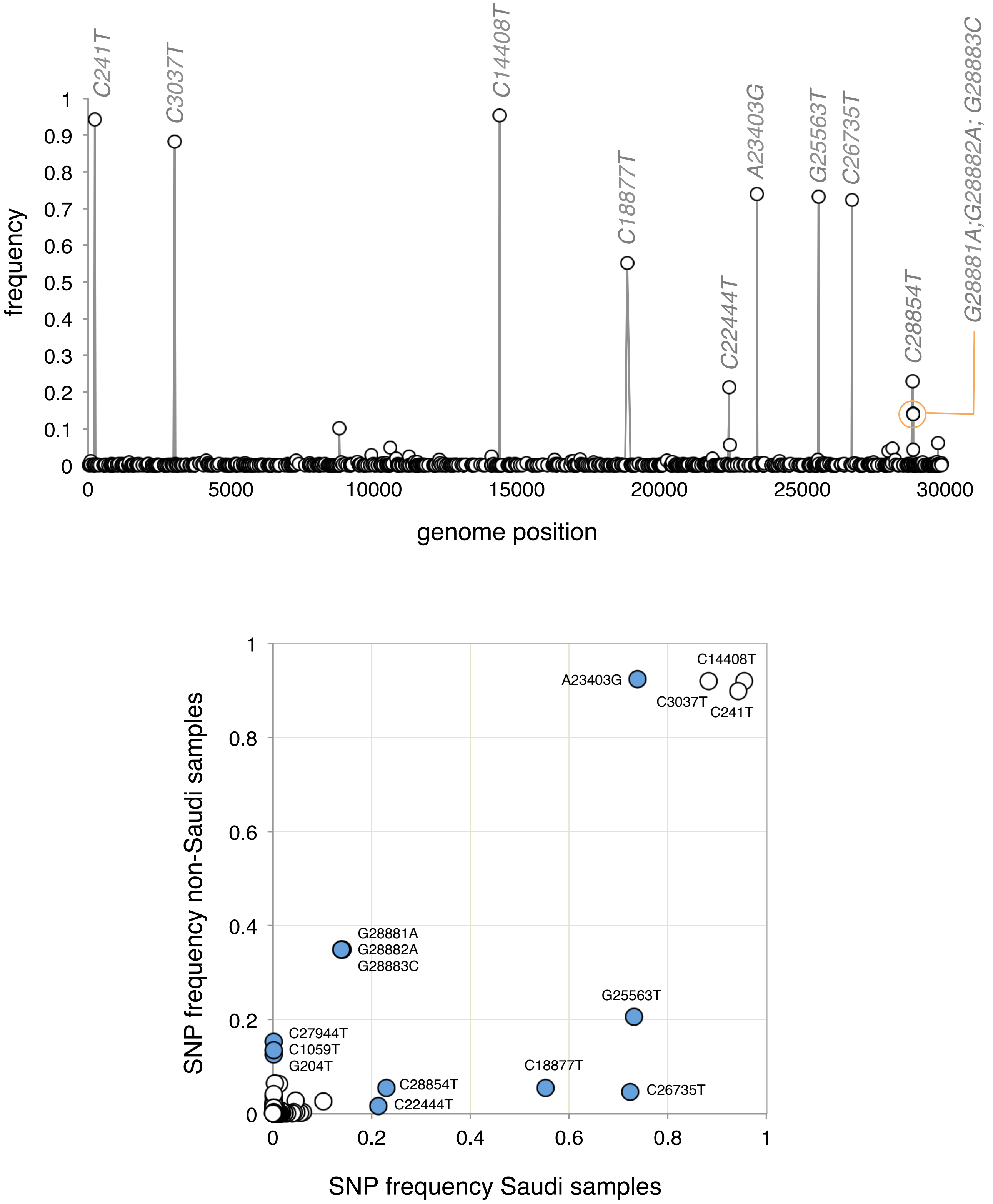

Supplementary Figure S2
Top: The 836 detected SNPs are shown along their positions in the SARS-CoV-2 genome (x-axis) and their frequency in the Saudi samples (y-axis).
High-frequency SNPs are highlighted along with the 3 SNPs underlying the R203K/G204R changes in the N protein (G28881A;G2882A;G28883C).
Bottom: Scatter plot of SNP frequencies in Saudi samples (y-axis) and in global, non-Saudi samples available from GISAID in 2020. SNPs differing by at least 0.1 in absolute values are highlighted in blue.

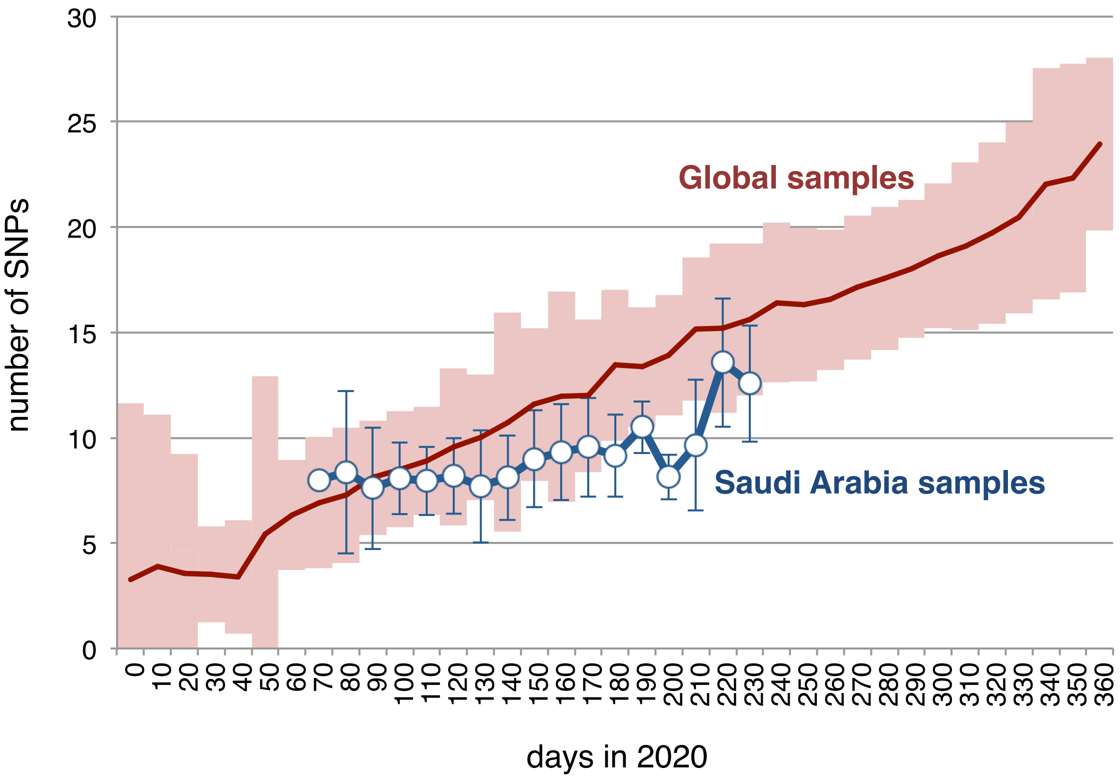

Supplementary Figure S3

Samples were grouped into 10-days periods according to their sampling date. The number of SNPs (compared to the Wuhan-Hu-1 reference) was recorded. Within each time group, the average number of SNPs and the standard deviation were then calculated.

Average SNPs in global samples – excluding Saudi Arabia –are shown as red line, with boxes showing plus/minus one standard deviation. Average SNPs in samples from Saudi Arabia are shown as blue line with whiskers denoting plus/minus one standard deviation.

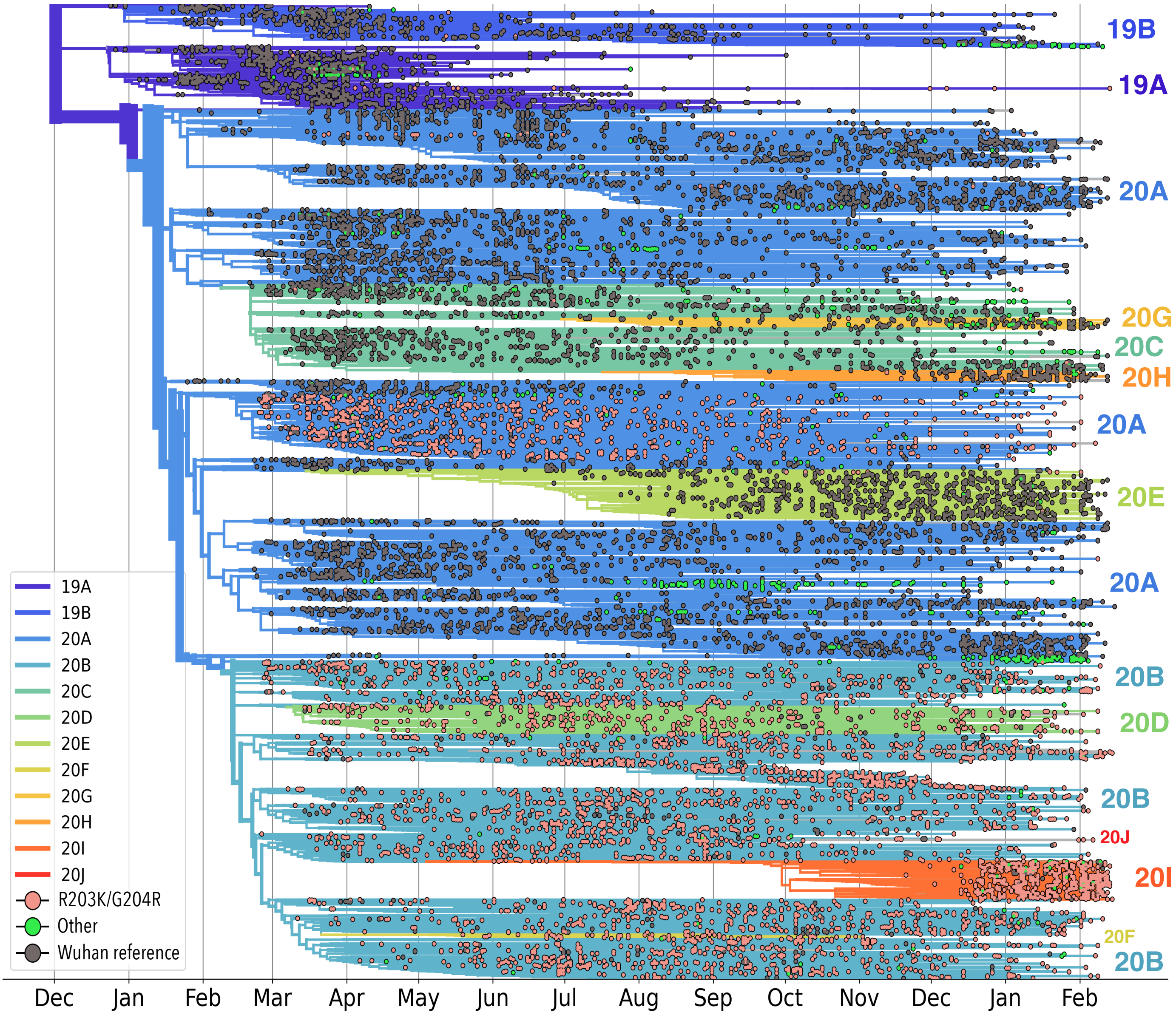

Supplementary Figure S4
ML tree of 16,386 sequences dated with TreeTime showing the distribution of genotypes at genome positions 28,881-28,883 Samples are coloured according to their genotype and branches according to Nextstrain clades. R203K/G204R SNPs were identified from 590K samples submitted to GISAID on February 24, 2021, and are all included in the shown subset.

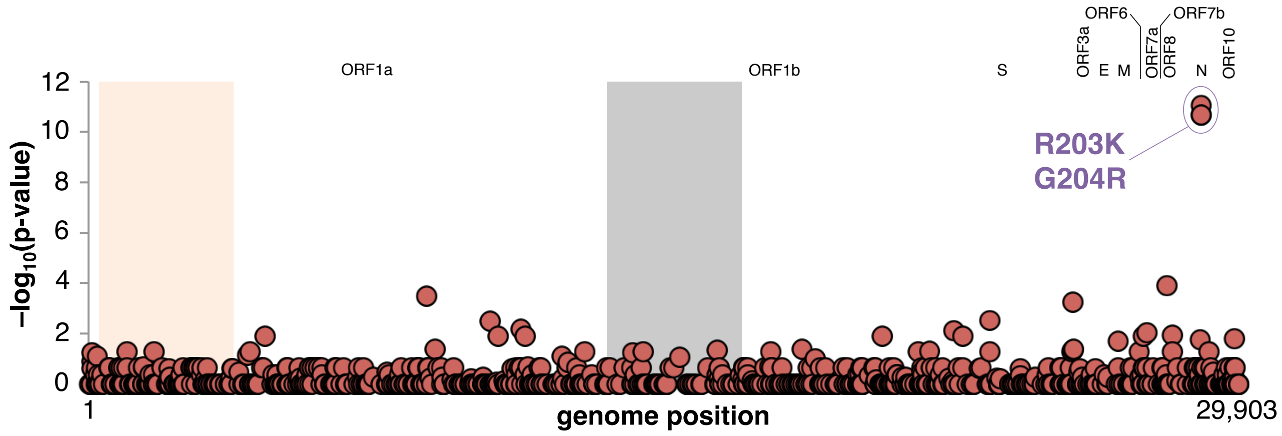

Supplementary Figure S5
Manhattan plot showing the association between SARS-CoV-2 SNPs and recorded mortality in 850 samples from Saudi Arabia with available mortality information. Negative $\log_{10}$(uncorrected p-values) from Fisher's exact tests are shown as red circles. Gene boundaries are indicated by background colors (listed on top), and the three R203K/G204R SNPs (positions 28,881-28,883) in the N gene are highlighted.

SNP	Count
GGC	23
GAG	47
GAA	29
AGG	563
AGC	27
AAG	26
AAC	232,475

Clade	R203K/G204R counts
19A	22
19B	4
20A	523
20A.EU2	30
20B	114,487
20C	12
20D	5,671
20E (EU1)	15
20F	12,593
20G	2
20H/501Y.V2	5
20I/501Y.V1	98,552
20J/501Y.V3	245

Supplementary Figure S6
Global samples from GISAID (February 24th 2021).
Left: Counts of partial and full R203K/G204R SNPs at genome position 28,881-28,883, where the Wuhan reference has the GGG genotype.
Right: Counts of R203K/G204R SNPs in different Nextstrain clades.

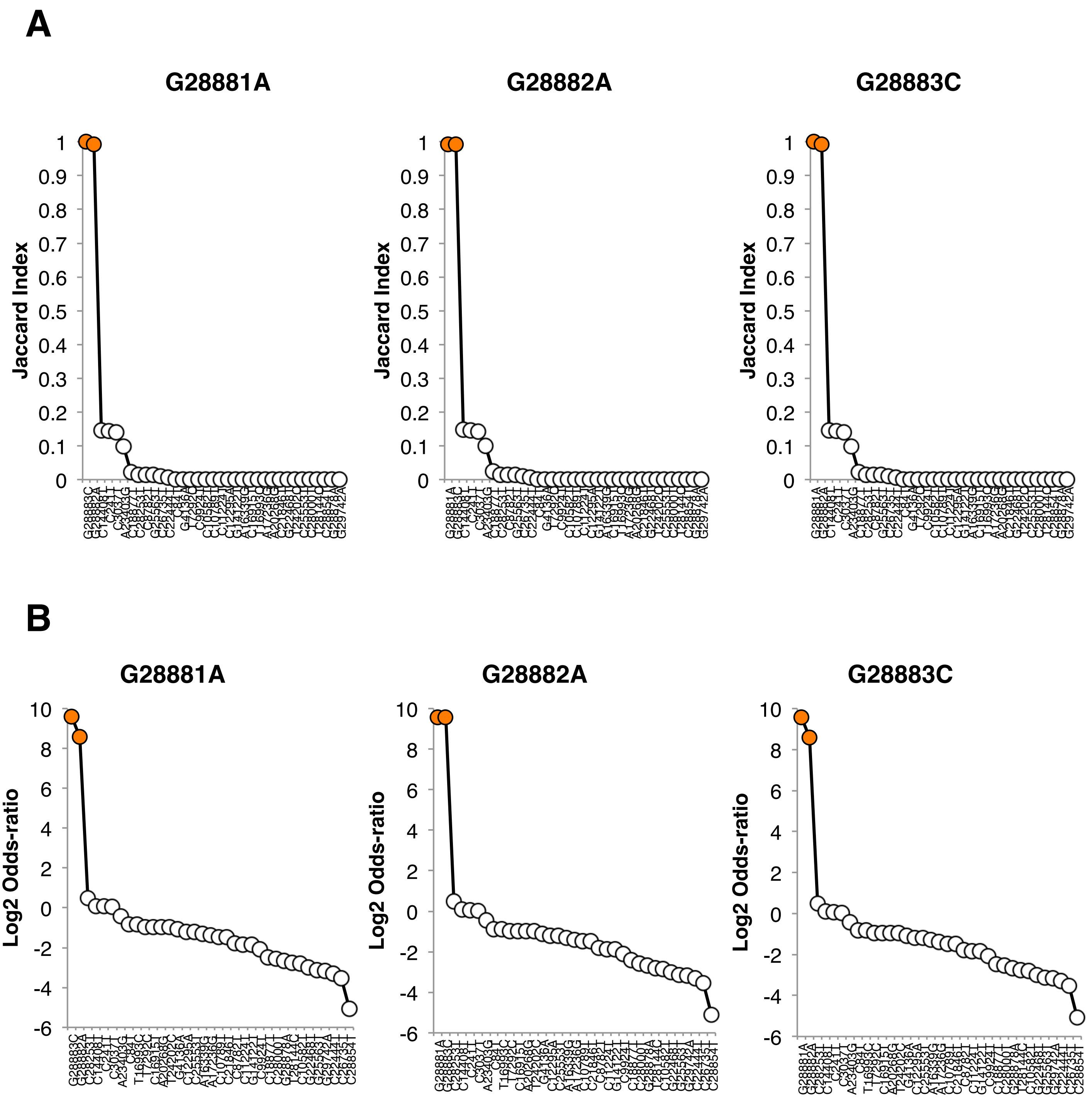

Supplementary Figure S7
Co-occurrences between the R203K/G204R SNPs and other SNPs in 892 SARS-CoV-2 genomes from Saudi Arabia shown as Jaccard Index (A) and log2 odds-ratio (B). The co-occurrence between the three SNPs in the R203K and G204R mutations (genomic mutations shown above plots) and all SNPs present in at least 20 samples (x- axes) are shown as circles. Co-occurrences between the three SNPs (G28881A, G28882A, and G28883C) are highlighted in orange.

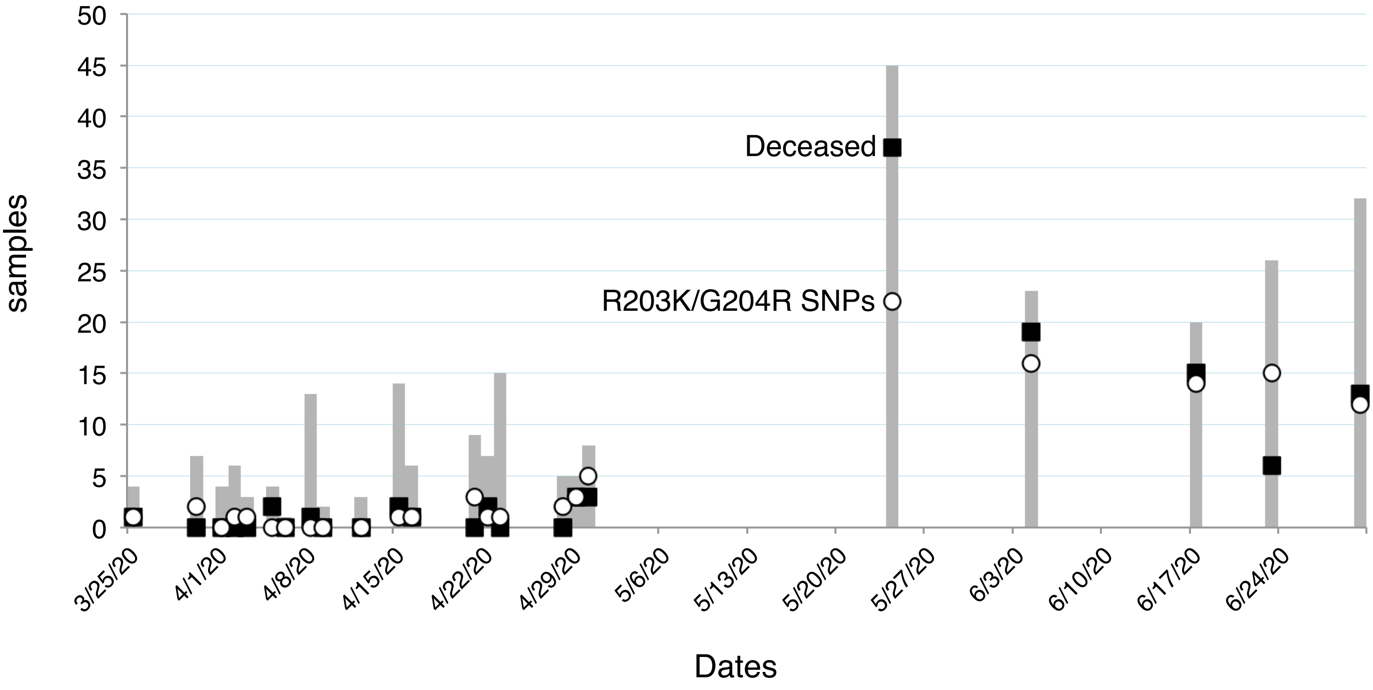

Supplementary Figure S8
Bar chart showing the number and collection dates of samples from King Abdullah Medical Centre in Jeddah. The number of samples from deceased patients are shown as black squares on the bars, and the number of samples containing the R203K/G204R SNPs shown as open circles.

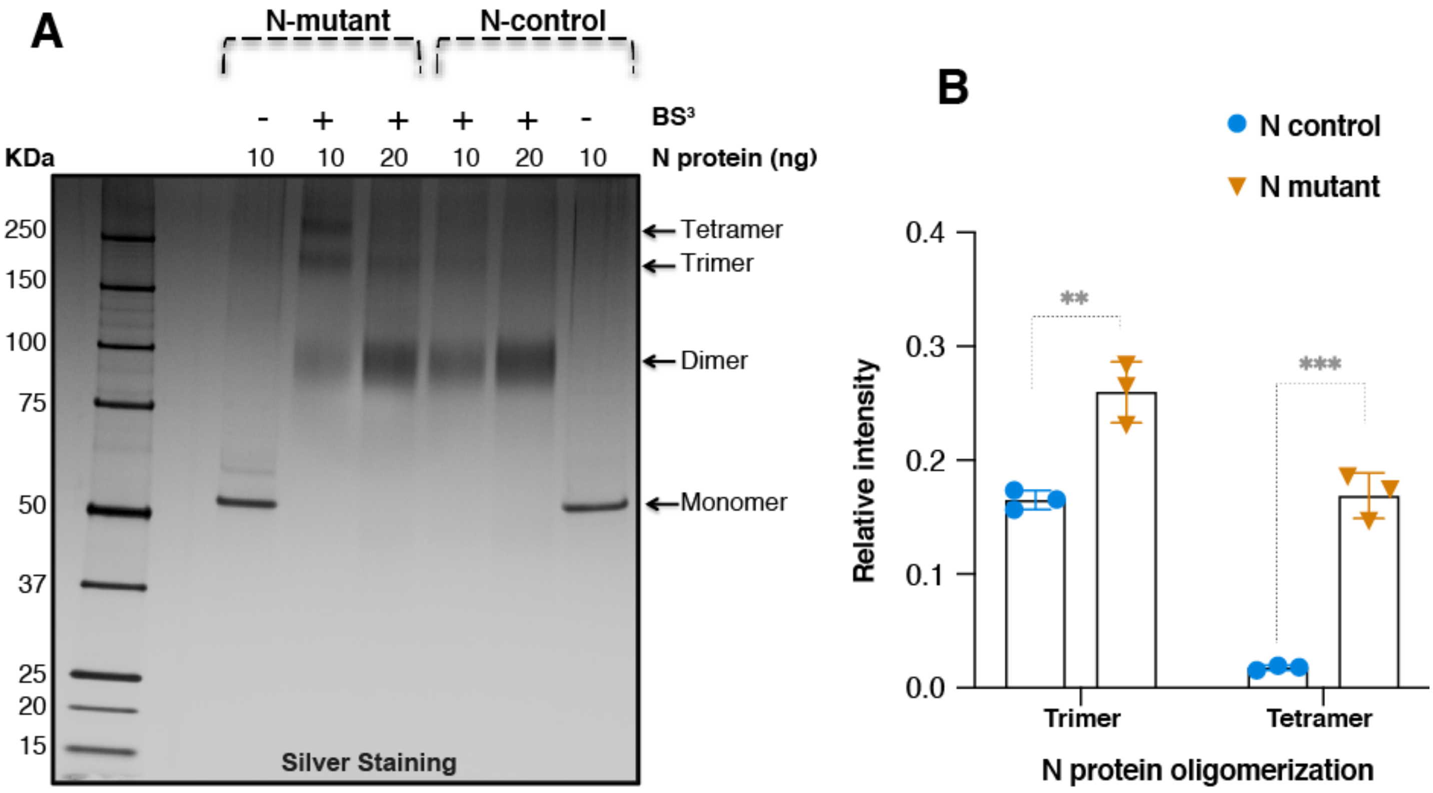

Supplementary Figure S9

Oligomerization analysis of mutant and control N protein. A) BS^3 cross-linking (2mM) and SDS-PAGE analysis of the oligomerization forms of mutant and control N proteins. Proteins were separated on SDS-PAGE and subjected to silver staining. B) Densitometry analysis of bands corresponding to oligomeric forms (trimer and tetramer) was performed. Bar-plot represents the relative intensities from three independent experiments (as shown mean ± SD). (Unpaired two-sided t-test, p values (0.000203***) and (0.00427**).

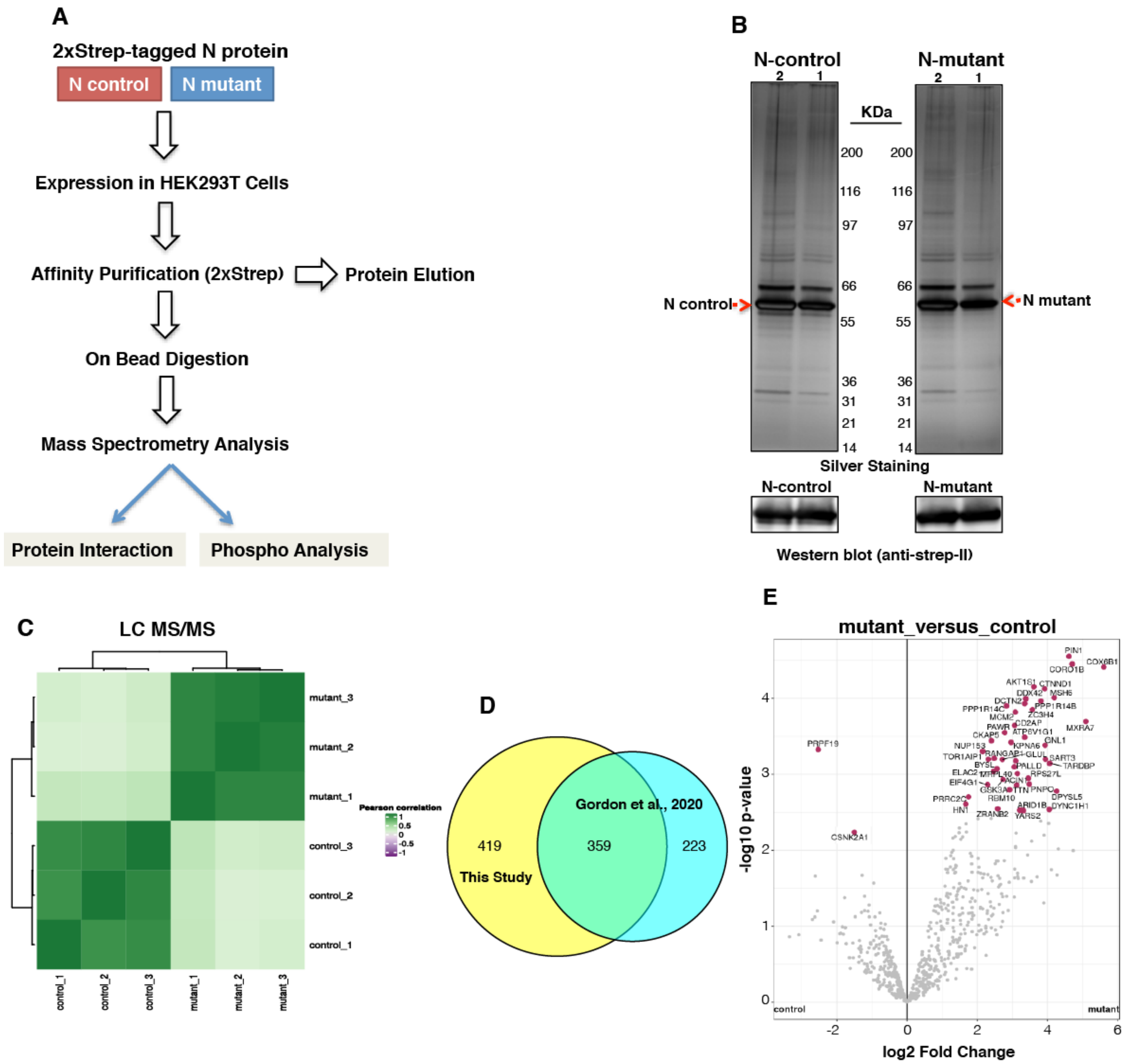

Supplementary Figure S10

Affinity mass spectrometry (AP-MS) analysis of mutant and control SARS-CoV-2 N protein and host protein interaction. A) Sketch showing the workflow of affinity mass spectrometry procedure. HEK-293 cell expressing 2XStrep-tagged control and mutant N protein were used for MagStrep affinity purification. Purified proteins were separated on SDS-PAGE and subjected to silver staining and western blotting for confirmation. After confirmation, interacting proteins were analyzed by mass spectrometry. B) (Upper) Silver staining of control and mutant N protein associated host proteins (1 and 2 show two loading volume), n=3 independent experiments were performed. (Lower) Western blot confirmation of N protein (mutant and control) using anti-Strep antibody. A full scan is available as Figure S12. C) Correlation matrix of three replicates for control and mutant N protein AP-MS. D) Overlapping of identified N interacting proteins with N-interacting proteins reported in previous study[27] (Gordon et al., 2020 *Nature*). E) Volcano plot displaying the differential interactions of pairwise comparisons (mutant_vs_control) in – Log10 adj. p-values vs. the Log2 protein fold change. Proteins with statistically significant (Adjusted p-value <= 0.05, and Log fold change >= 1) difference between mutant and control AP-MS conditions are highlighted.

Supplementary Figure S11

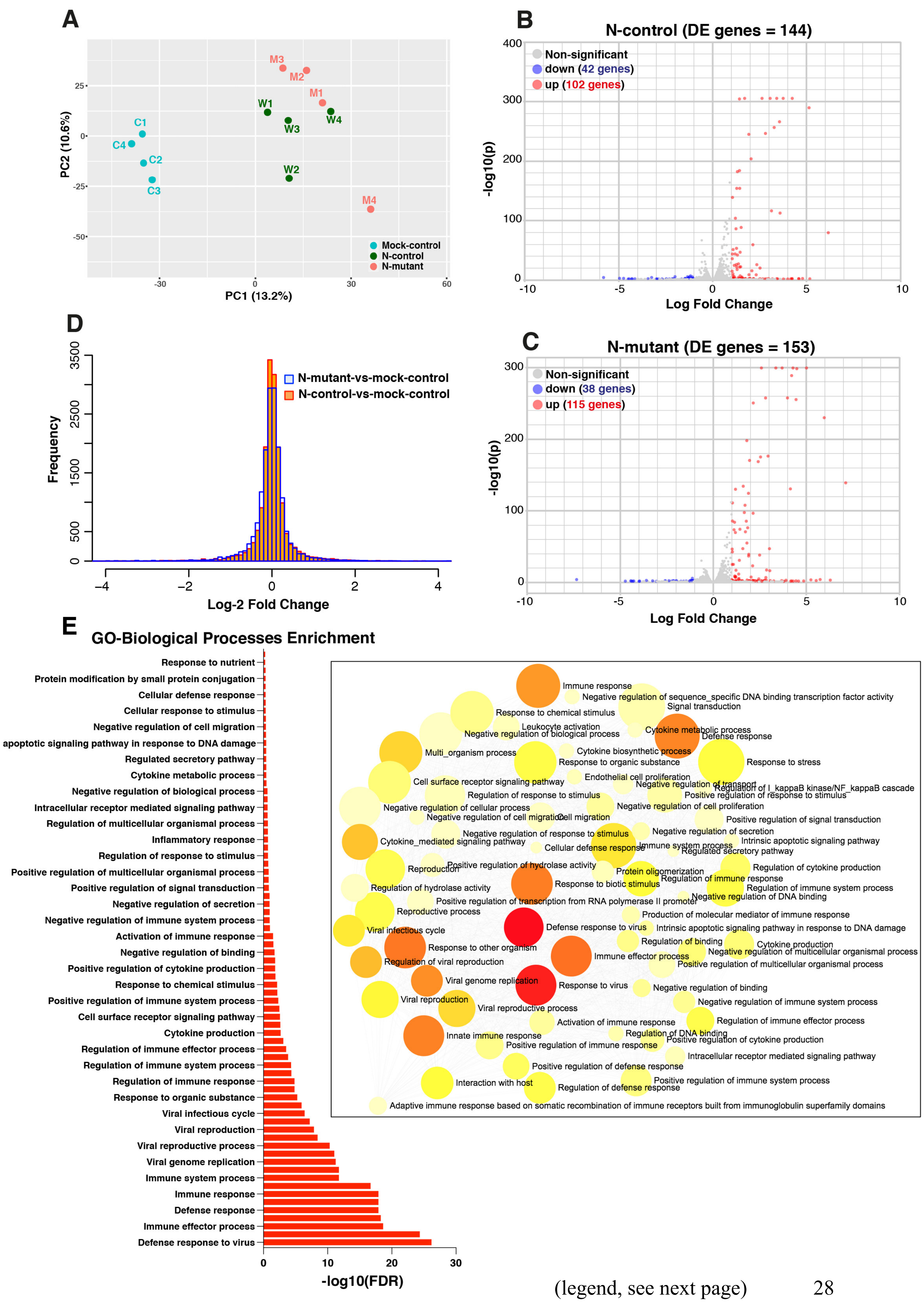

(legend, see next page)

Supplementary Figure S11. Transcriptomic analysis of mutant and control N transfected host cells.

A) PCA on transcriptome of Calu-3 cells transfected with plasmids expressing the full-length N-control and N-mutant protein along with mock control. **B-C**) Volcano-plot showing differentially expressed (DE) genes based on a filtering criterion of adj p-value < 0.05 and fold-change cutoff ≥ 1) as determined by the method EdgeR in NetworkAnalyst tool. X-axis depicts log2 fold-change of DE genes and Y-axis depicts –log10 P-value. Genes with significant up-regulation are shown in red and down-regulated are shown in blue. All other non- significant genes are shown in gray. **D**) Plot showing the distribution of log2-fold changes in both N-mutant and N-control conditions. **E**) GO enrichment analysis of all DE genes in the N-mutant condition. The enriched GO BP (Biological Processes) terms are displayed by plotting against the –log10 of the false discovery rate (FDR q value). The enriched terms display an interconnected network with overlapping gene sets (from the list). Each node represents an enriched term and colored by its FDR q value (red color shows the lowest values as shown in the bar-chart and Supplementary Data 6). The size of each node corresponds to number of linked genes from the list.

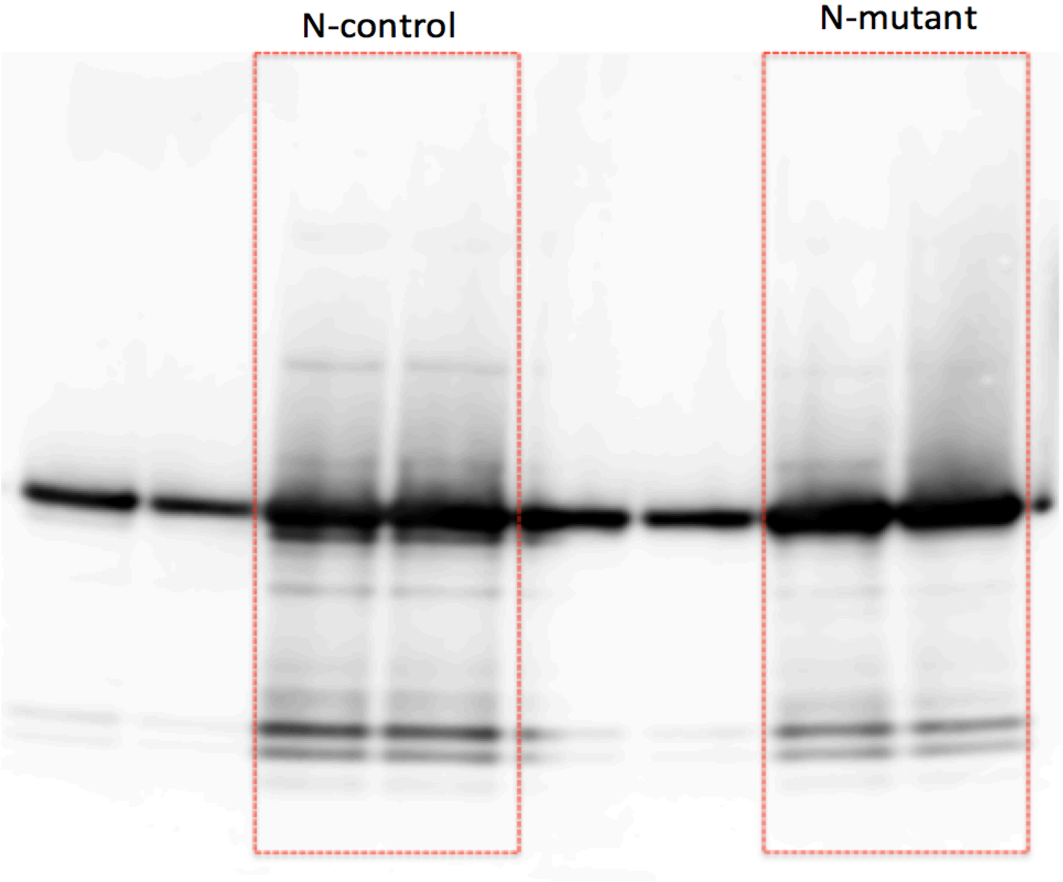

Supplementary Figure S12
Full scan for Supplementary Figure S10b (lower panel)
