# Supplementary material for: SARS-CoV-2 genomes from Saudi Arabia implicate nucleocapsid mutations in host response and increased viral load
Source: Nat Commun. 2022 Feb 1;13:601. doi: 10.1038/s41467-022-28287-8 (PMC8807822; doi:10.1038/s41467-022-28287-8)
Supplement: Supplementary file 3 — Description of Additional Supplementary Files [file 41467_2022_28287_MOESM3_ESM.pdf]

Description of Additional Supplementary Files

File Name: Supplementary Data 1
Description: Sample names and ID along with GISAID accession numbers

File Name: Supplementary Data 2
Description: Identified protein groups. A) N protein (N control and mutant AP-MS. B) Mock AP-MS

File Name: Supplementary Data 3
Description: List of proteins differentially interacting with mutant and control N proteins. Both significant and non-significant proteins are listed.

File Name: Supplementary Data 4
Description: Phosphorylated sites detected in N-control and N-mutant proteins

File Name: Supplementary Data 5
Description: Table showing all differentially expressed genes. Differential expression analysis was performed using EdgeR integrated in NetworkAnalyst.

File Name: Supplementary Data 6
Description: Table showing overrepresentation analysis (ORA) of pathways significantly overlapped with the differentially expressed genes by NetworkAnalyst tool. Hypergeometric tests are used to compute the p-values.

File Name: Supplementary Data 7
Description: Proteins detected in N protein (mutant and wildtype) and Mock AP-MS.
