# Supplementary material for: SARS-CoV-2 genomes from Saudi Arabia implicate nucleocapsid mutations in host response and increased viral load
Source: Nat Commun. 2022 Feb 1;13:601. doi: 10.1038/s41467-022-28287-8 (PMC8807822; doi:10.1038/s41467-022-28287-8)
Supplement: Supplementary file 11 — Reporting Summary [file 41467_2022_28287_MOESM11_ESM.pdf]

nature research

Corresponding author(s): Arnab Pain

Last updated by author(s): Nov 30, 2021

Reporting Summary

Nature Research wishes to improve the reproducibility of the work that we publish. This form provides structure for consistency and transparency in reporting. For further information on Nature Research policies, see our Editorial Policies and the Editorial Policy Checklist.

Statistics

For all statistical analyses, confirm that the following items are present in the figure legend, table legend, main text, or Methods section.

n/a	Confirmed	
☐	☒	The exact sample size (*n*) for each experimental group/condition, given as a discrete number and unit of measurement
☐	☒	A statement on whether measurements were taken from distinct samples or whether the same sample was measured repeatedly
☐	☒	The statistical test(s) used AND whether they are one- or two-sided<br>*Only common tests should be described solely by name; describe more complex techniques in the Methods section.*
☐	☒	A description of all covariates tested
☒	☐	A description of any assumptions or corrections, such as tests of normality and adjustment for multiple comparisons
☐	☒	A full description of the statistical parameters including central tendency (e.g. means) or other basic estimates (e.g. regression coefficient) AND variation (e.g. standard deviation) or associated estimates of uncertainty (e.g. confidence intervals)
☐	☒	For null hypothesis testing, the test statistic (e.g. *F*, *t*, *r*) with confidence intervals, effect sizes, degrees of freedom and *P* value noted<br>*Give P values as exact values whenever suitable.*
☒	☐	For Bayesian analysis, information on the choice of priors and Markov chain Monte Carlo settings
☒	☐	For hierarchical and complex designs, identification of the appropriate level for tests and full reporting of outcomes
☒	☐	Estimates of effect sizes (e.g. Cohen's *d*, Pearson's *r*), indicating how they were calculated

Our web collection on statistics for biologists contains articles on many of the points above.

Software and code

Policy information about availability of computer code

Data collection	Image lab software version 5.2.1 (build 11)
Data analysis	Data analysis used the following softwares: bcftools (v1.9), BWA (v0.7.17), GATK (v4.1.7), IQ-TREE (v1.6.12 & v2.0.5), kallisto (v0.43.1), minimap2 (v2.17), picard tools (v2.20.4), R (v4.0.3), R package ape (v5.5), R package phangorn (v2.7.0), R package ggplot2 (v3.3.5), R package treedater (v0.5.0), R package Skygrowth (v0.3.1), R package sarscov2 (v0.1.4), R package mgcv (v1.8.33), samtools (v1.8), tn93 (v1.0.6), TreeTime (v0.7.5), Trimmomatic (v0.38), Maxquant (v1.5.3.30), NetworkAnalyst (v3.0), GraphPad Prism 9 (v9.1.1), and LFQ-analyst (v1.0) tools.

For manuscripts utilizing custom algorithms or software that are central to the research but not yet described in published literature, software must be made available to editors and reviewers. We strongly encourage code deposition in a community repository (e.g. GitHub). See the Nature Research guidelines for submitting code & software for further information.

Data

Policy information about availability of data

All manuscripts must include a data availability statement. This statement should provide the following information, where applicable:

- Accession codes, unique identifiers, or web links for publicly available datasets
- A list of figures that have associated raw data
- A description of any restrictions on data availability

Supplementary Information is available for this paper. Assembled virus genomes are available at GISAID (Supplementary Table S1). Raw viral reads, assembled virus genomes, and reads from RNA-Seq analysis of transfected Calu-3 cells have been uploaded to European Nucleotide Archive under the Study accession number PRJEB45515 [https://www.ebi.ac.uk/ena/browser/view/PRJEB45515].
The relevant source data for figures 1, 3, 4, and 5, as well as supplementary figures S1, S2, S3, S5, S7, S8, S9, S10, S11, and S12 are provided with this paper.

The affinity-purification mass spectrometry (AP-MS) proteomics data have been deposited to the ProteomeXchange Consortium via the PRIDE partner repository with the dataset identifier PXD027168 [http://proteomecentral.proteomexchange.org/cgi/GetDataset?ID=PXD027168].

Field-specific reporting

Please select the one below that is the best fit for your research. If you are not sure, read the appropriate sections before making your selection.

☒ Life sciences ☐ Behavioural & social sciences ☐ Ecological, evolutionary & environmental sciences

For a reference copy of the document with all sections, see nature.com/documents/nr-reporting-summary-flat.pdf

Life sciences study design

All studies must disclose on these points even when the disclosure is negative.

Sample size	During a six months period (March to August 2020), available nasal swab samples from COVID-19 patients were collected from eight hospitals and one quarantine hotel. For sequencing, we used all obtained samples with sufficient Ct values (below 35), A total of 892 q-RT-PCR confirmed samples of SARS-COV-2 were successfully used for whole genome sequencing.
Data exclusions	Samples with Ct values above 38 were omitted from genome sequencing, and only samples (892) from which a complete viral genome could be assembled were kept for further analysis. This exclusion criteria was pre-established.
Replication	For all experimental works three or four biological replicates and technical duplicates were used. We confirmed that all attempts at replication were successful.
Randomization	All samples with adequate viral sequence material were used for viral genome assembly.
Blinding	Blinding was not relevant in this study as samples were de-identified at the point of receipt and metadata was only considered after the assembly of viral genomes.

Reporting for specific materials, systems and methods

We require information from authors about some types of materials, experimental systems and methods used in many studies. Here, indicate whether each material, system or method listed is relevant to your study. If you are not sure if a list item applies to your research, read the appropriate section before selecting a response.

Materials & experimental systems

n/a	Involved in the study	
☐	☒	Antibodies
☐	☒	Eukaryotic cell lines
☒	☐	Palaeontology and archaeology
☒	☐	Animals and other organisms
☐	☒	Human research participants
☒	☐	Clinical data
☒	☐	Dual use research of concern

Methods

n/a	Involved in the study	
☒	☐	ChIP-seq
☒	☐	Flow cytometry
☒	☐	MRI-based neuroimaging

Antibodies

Antibodies used	The anti-strep-II antibody (abcam, ab76949) was used for western blot.
Validation	This antibody specificity is species independent according to manufacturer. This antibody has been validated for western-blot by the company (abcam).

Eukaryotic cell lines

Policy information about cell lines

Cell line source(s)	Calu-3 cell line (ATCC; HTB-55) was used in this study.
Authentication	The cell line was not authenticated
Mycoplasma contamination	Calu-3 cell line was tested for mycoplasma contamination upon receipt and periodically thereafter. They tested negative for mycoplasma contamination.

Commonly misidentified lines (See ICLAC register)	No misidentified lines used in this study

Human research participants

Policy information about studies involving human research participants

Population characteristics	We obtained deidentified nasopharyngeal swab samples from COVID-19 patients from eight hospitals and one quarantine hotel. The information about samples including age, gender, nationality are shown in Figure S1, and other information such as disease outcome, genotype of the infecting virus, and comorbidities are provided in Table S2-S3.
Recruitment	All provided nasopharyngeal swab samples from COVID-19 patients were used in our study.
Ethics oversight	Ethical approvals were obtained from the Institutional review board of the Ministry of Health in Makkah region with the numbers H-02-K-076-0420-285 and H-02-K-076-0320-279, as well as the Institutional review board of Dr. Sulaiman Al Habib Hospital number RC20.06.88 for samples from Riyadh and the Eastern regions respectively.

Note that full information on the approval of the study protocol must also be provided in the manuscript.
